# Supplementary figures and images for: A transformer-based deep learning framework to predict employee attrition
Source: PeerJ Comput Sci. 2023 Sep 27;9:e1570. doi: 10.7717/peerj-cs.1570 (PMC10557501; doi:10.7717/peerj-cs.1570)

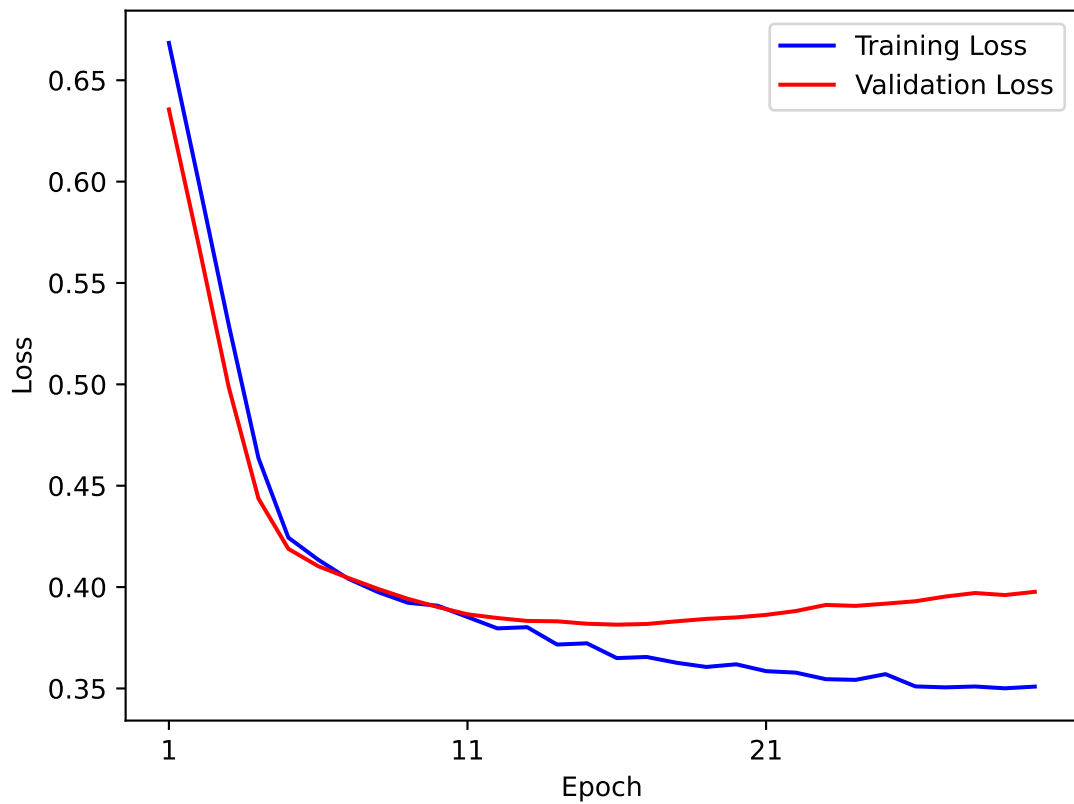

Supplement: Supplemental Information 1 [file peerj-cs-09-1570-s001.zip › output/loss.pdf]
